# The Relationship between High-Intensity Physical Activity and Traumatic Dental Injury among Young Adults in South Korea

**DOI:** 10.1155/2024/9678841

**Published:** 2024-06-10

**Authors:** Ji-Young Son, Dong-Hun Han

**Affiliations:** ^1^ Department of Preventive and Social Dentistry School of Dentistry Seoul National University, 1, Gwanak-ro, Guanak-gu, Seoul 08826, Republic of Korea; ^2^ Dental Research Institute Seoul National University, Seoul, Republic of Korea

## Abstract

**Materials and Methods:**

This study analyzed data collected from the 5th National Health and Nutrition Examination Survey (KNHANES V:2010−2012). The total number of participants in the 5th KNAHANES was 5,383 young adults aged 19–39 years, selected from 25,534 participants. Logistic regression analysis was performed using socioeconomic status (sex, age, education level, and income), physical activity intensity (vigorous and moderate), frequency of vigorous and moderate physical activity (days per week), and traumatic dental injuries due to exercise.

**Results:**

A total of 5,383 participants were included in the analysis. High-intensity exercisers had a statistically different association with traumatic dental injuries due to exercise. In all models, high-intensity exercisers had more traumatic dental injuries than moderate-intensity exercisers, and participants who exercised vigorously 4 or more days per week had a significantly higher prevalence experience of traumatic dental injuries. Among adults in their 20s, men, college attendees, and those with higher incomes, the prevalence of exercising vigorously 4 or more days per week was higher.

**Conclusions:**

Among young adults, a higher frequency of high-intensity physical activity was associated with a higher prevalence experience of traumatic tooth injury due to exercise compared with no physical activity.

## 1. Introduction

In modern society, young adults are increasingly concerned about their health and leading active lifestyles. Various extreme sports and recreational activities have gained popularity, and many young adults are known to energize their lives with hobbies and leisure activities [1]. It has been demonstrated that high-intensity exercise, such as running, leapfrog, mountain climbing, fast cycling, and fast swimming, among others, enhances the function of the human body. However, it is more likely to lead to muscle damage [2]. High-contact sports and team activities may increase the prevalence of traumatic dental injury (TDI) due to vigorous physical contact. Several studies have investigated sports-related TDI [3, 4]. with a high prevalence of dental trauma due to collisions or falls during training and games, especially in sports with excessive physical contact such as soccer and basketball [5]. Also, with the recent increase in popularity of these high-intensity sports, there is an increased risk of unintentional trauma [6]. Trauma is caused by a sudden external impact or force, and the International Health Organization reports that 58 million people die from trauma each year, accounting for 9% of all deaths worldwide [7]. This shows how serious a problem trauma is for people.

Among physical trauma, head and neck trauma, which accounts for half of all trauma, is often associated with TDI [8]. Damage to teeth, especially those with masticatory, articulatory, and esthetic functions, directly affects daily life. When the functional side of the tooth is compromised, it reduces the individual's quality of life. TDI can result in long-term sequelae and discoloration due to the materials used in the treatment, which can lead to a decrease in social adaptations such as self-confidence and speaking ability [9]. In addition, dental trauma has a significant negative esthetic impact as it occurs primarily in the maxillary central incisors [10].

It is known that high-intensity exercise is performed under extreme conditions, which increases the risk of traumatic injury [11]. Traumatic musculoskeletal fractures, contusions, and head and neck injuries can impair physical function [2, 12] and limit daily activities, making it difficult for young people to maintain an active lifestyle. Recent studies have also shown that various types of trauma can have negative psychological effects, such as depression and low self-esteem [13, 14, 15]. In addition, trauma can lead to problems with self-esteem, competence, mood, and social anxiety due to changes in appearance [16, 17].

Therefore, based on the characteristics of young adults who engage in vigorous and high-intensity exercise, this study aims to analyze the experience prevalence of exercise-induced TDI by comparing the frequency of vigorous and moderate physical activity and to identify socioeconomic risk factors to provide information that can help prevent exercise-induced TDI so that young adults can maintain an active lifestyle.

## 2. Materials and Methods

### 2.1. Study Population

This study used data from the 5th Korea National Health and Nutrition Examination Survey (KNHANES V). All procedures contributing to this study complied with the ethical standards of relevant national and institutional human experimentation committees and the Declaration of Helsinki of 1975, as revised in 2008. All procedures were approved by the Institutional Review Board of the Korean Centers for Disease Control and Prevention, and all participants signed written informed consent.

The total number of participants in the fifth wave of KNAHANES was 5,383 young adults aged 19–39 years old, selected from a total of 25,534 participants. Missing data were excluded, and only those who completed a questionnaire about their TDI experience were included. We compared the demographics and physical activity frequency and intensity of all subjects (*n* = 5,383) according to whether they had experienced a TDI.

KNHANES was conducted as a general survey representative of the noninstitutionalized civilian population of South Korea. This population-based survey included three assessments, health interviews and examinations and a dietary survey, and used a complex multistage sampling design. The study was approved by the Institutional Review Board of the CMC Clinical Research Coordinating Center (IRB approval number: KC22ZADI0486).

### 2.2. Outcome and Explanatory Variables

The National Health and Nutrition Examination Survey asks about the experience of TDI due to exercise or accident. Participants who answered “yes” were asked more detailed questions about the cause of the injury: (i) exercise; (ii) violence; (iii) traffic accidents; and (iv) safety-related accidents. The outcome variable for this study was defined as “TDI due to exercise.” Covariates included sociodemographic and socioeconomic factors. Sociodemographic factors included age (continuous) and sex (categorical: male and female). Socioeconomic factors included personal income and education, representative of the literature on their association with physical activity [18, 19]. Personal income was categorized into first, second, third, and fourth quintiles, and educational attainment was categorized into three groups: less than a high school diploma, never attended college/not in college, and college graduate/more than a high school diploma. The causal variables in this study were exercise intensity and frequency. Exercise intensity was categorized as “vigorous” and “moderate.” According to the survey, vigorous physical activity was defined as “In the past week, on how many days did you do vigorous physical activity for 10 min or more that made you feel very tired or out of breath more than usual?” This includes activities such as running (jogging), mountain climbing, fast cycling, fast swimming, soccer, basketball, jumping rope, squash, singles tennis, and heavy lifting. The survey defined moderate physical activity as “In the past week, on how many days did you do moderate physical activity for 10 min or more of that made you feel very tired or out of breath more than usual?” This includes occupational and physical activities such as slow swimming, doubles tennis, volleyball, badminton, table tennis, and light lifting but excluded walking. The frequency of both vigorous and moderate physical activity was measured in “days per week.”

### 2.3. Statistical Analysis

Descriptive characteristics of the study population were calculated from the sample data using weighted percentages (%) and 95% confidence intervals (CI) to identify differences in socioeconomic status, health behaviors, and health status among participants. Because KNHANES uses a complex sampling design, we analyzed the data using complex sampling methods, including stratification, weighting, and primary sampling units. Chi-squared tests were used to identify differences in exercise-induced TDI by socioeconomic status and exercise intensity and frequency. Bivariate logistic regression was used to determine the odds ratios of TDI by frequency of vigorous versus moderate physical activity after adjustment for potential confounders. Regression Model 1 is the crude model, and sociodemographic variables (sex and age) were added in regression Model 2. The socioeconomic level variable, income level, was added in regression Model 3. Finally, educational level was added in regression Model 4. Statistical significance was defined as a *p* value <0.05. All data collection and statistical analyses were performed using SPSS version 23.0 (IBM, Armonk, New York, USA).

## 3. Results

The study included 5,383 young adults between the ages of 19 and 39, comprising 2,197 males and 3,186 females. Each variable had different missing values, resulting in distinct total numbers for each.  Table 1 shows the TDI due to exercise attributed to general characteristics, intensity, and frequency of exercise. The mean age of young adults who experienced TDI due to exercise was weighted at 27.20 years. The male participants and those who had a high school education or less were more likely to experience TDI due to exercise. Statistically significant differences in TDI experience were observed based on sex, age, and education and diabetes mellitus. Although TDI experience was higher in the highest income group, smoking group, and drinking group, the difference was not statistically significant. 2.3% of participants who exercised vigorously on 4 or more days experienced TDI, and the prevalence decreased as the number of days of exercise decreased. The prevalence experience of TDI significantly varied based on the frequency of vigorous exercise. However, participants who exercised moderately on 4 or more days experienced TDI at a rate of 1.6%, which was not statistically significant.

The results of a logistic regression model of TDI experience as a function of exercise intensity and frequency are presented in Table 2. In this study, Model 1 is used as the reference model for comparing with Models 2, 3, and 4. For Model 1, participants who exercised vigorously for 4 or more days per week were 4.81 times (with a 95% confidence interval of 1.55–14.89) more likely to experience a TDI compared to those who did not. After adjusting for sex and age as sociodemographic characteristics, the TDI experience was 3.16 times (with a 95% confidence interval of 1.04–9.57) higher for participants who exercised 4 or more days per week in comparison with those who never exercised vigorously in Model 1. In Model 3, after including income level as a proxy for socioeconomic characteristics to Model 2, the TDI experience was 3.35 times (with a 95% confidence interval of 1.03–10.86) higher compared to Model 1. However, for Model 4, after adding education as a variable to Model 3, the TDI experience was 2.41 times (with a 95% confidence interval of 0.67–8.71) higher compared to Model 3, but this difference was not statistically significant. Participants who exercised 4 or more days per week had a 3.11 times higher likelihood (1.02–9.46) of experiencing TDI in Model 1 when compared to those who did not exercise moderately. This difference was statistically significant. Sociodemographic characteristics added to Model 1 reduced the odds ratio to 2.38 (0.79–7.19). Income level added to Model 2 raised the odds ratio to 2.52 (0.79–8.08), and adding education to Model 3 raised the odds ratio to 1.84 (0.52–6.45). However, this difference was not statistically significant.

Table 3 presents the frequency of intense exercise based on general characteristics. Men were twice as likely as women to engage in intense exercise 4 or more days a week. Additionally, they tended to engage in intense exercise 2–3 days a week and once a week. Those who engaged in intense exercise 4 or more days a week were more likely to have graduated from high school, and in the highest income group, and were statistically significant. In contrast, people who never engaged in intense exercise were more likely to be women, college graduates, and have lower incomes. In addition, those who engaged in vigorous exercise 4 or more days a week were higher and statistically significant in the smoking group and higher but not statistically significant in the drinking group. Statistically significant associations existed between gender, age, education, income, smoking status, and the frequency of intense exercise.

## 4. Discussion

This study examined the prevalence of TDI among young adults who participated in vigorous versus moderate physical activity. Young adults who engaged in vigorous and moderate physical activity each had a TDI experience rate of 0.7%. Compared with those who did not engage in vigorous physical activity, those who engaged in vigorous physical activity on 4 or more days were 4.81% more likely to experience TDI, and those who engaged in moderate physical activity on 4 or more days were 3.11% more likely to experience TDI. This suggests that the intensity and frequency of physical activity in young adults (aged 19–39 years) is associated with exercise-induced TDIs. It also shows that vigorous exercise is associated with higher TDI experiences compared to moderate exercise. In addition, a sequential increase in TDI experience was observed as the frequency of vigorous exercise increased.

TDI were found in 25% of all soccer players, and slightly more TDIs were soccer-related (44%) than non-soccer-related injuries (39%) [20]. In contrast, a large number of TDIs (39%) occurred within sports clubs. In addition, 80.6% of professional players and 37.7% of amateur players experienced oral soft tissue lacerations and fractures during basketball practice [21]. These studies emphasize that high-intensity activities may increase the risk of TDI. Our findings are consistent with studies showing that temporomandibular joint (TMJ) disorders increase with increasing exercise intensity [22] and hamstring injury rates increase [23]. It is also consistent with studies that have reported associations with a variety of traumatic injuries, including increased shoulder injuries in CrossFit, a recently popular high-intensity physical activity [24, 25]. In is possible that the association between high-intensity exercise and TDI is related to the greater forces and impacts that occur during high-intensity exercise. However, in contrast to our results, previous studies have shown that vigorous physical activity reduces the risk of hip and all fractures by 1%–40%, which has been interpreted to mean that healthy people have a lower risk of fracture because they exercise more [26].

Notably, young adults who participated in vigorous physical activity 4 or more days per week had a significantly higher prevalence of TDI than those who did not exercise at all. Even after adjusting for sex and age, the prevalence of TDI was 3.16 times higher among those who participated in vigorous exercise 4 or more days per week, indicating that the relationship between frequency of vigorous exercise and TDI was not confounded by sex or age. In addition, after adjustment for the socioeconomic indicator of personal income level, young adults who engaged in vigorous physical activity 4 or more days per week had a slightly increased prevalence of exercise-induced TDI of 3.35 times that of those who never exercised, and the association remained significant. However, after adjusting for education in Model 4, the odds of experiencing an exercise-induced TDI were significantly reduced to 2.41 times and lost statistical significance. This finding is consistent with a previous study that found no association with socioeconomic status among children in England, Wales, and Northern Ireland [27]. Other studies have also found no association between socioeconomic indicators and the incidence of permanent dental trauma. However, this may be explained by the fact that children aged 9–14 years live in relatively safe environments in similar physical settings such as schools [28].

On the other hand, there are studies that have shown an increased risk of TDI with lower parental education and poorer economic status [29] that differ from our results. In our study, higher levels of education were associated with less vigorous exercise, whereas higher levels of income were associated with more vigorous exercise. In addition, we conducted a cross-tabulation analysis to determine the relationship between education and income and found that they were proportional. A higher percentage of young adults in their 20s (11%) and 30s (7.9%) engaged in vigorous exercise 4 or more days per week. On the other hand, a slightly lower percentage of young adults in their 20s (14.3%) engage in vigorous exercise 2–3 days per week than young adults in their 30s (15.7%). This shows that while the association between age and vigorous exercise is statistically significant, there is no clear pattern by age.

However, most associations of socioeconomic status with the incidence of TDI have been studied in children and adolescents with a high incidence of dental trauma [30, 31, 32, 33]. Our study focused on young adults aged 19–39 years and therefore included many individuals in their 20s and 30s who were still in school. The inverse relationship between educational level and vigorous exercise is likely due to the fact that we identified a specific younger age group, 19–39 years old. Therefore, our results are not directly comparable with previous studies. Future studies are needed to specifically identify the mechanisms by which age and socioeconomic factors influence vigorous exercise and the TDI.

Although this study used a large nationally representative sample of South Koreans, the results should be interpreted in light of several limitations of secondary data. First, the design is cross-sectional, which may make it difficult to identify causal relationships. Second, although self-reported oral health status is highly reliable [34, 35], respondents may be subject to subjectivity. To overcome these limitations, longitudinal studies that repeatedly follow the same subjects repeatedly to identify changes over time are recommended. Third, due to the nature of the secondary data, it was not possible to identify TDI types through clinical examinations nor could covariates for other known associated conditions such as malocclusion be identified. This should be considered for future research. Despite these limitations, this study identifies TDI experiences over a short period of time in the past 12 months and thus confirms the association of vigorous exercise with exercise-induced TDI experiences in young adults. Efforts should be made to incorporate these findings into the development of appropriate TDI prevention programs for young adults, tailored to their socioeconomic level and consistent with modern active lifestyles.

## 5. Conclusion

Among young adults, a higher frequency of high-intensity physical activity was associated with a higher prevalence experience of traumatic tooth injury due to exercise. Furthermore, adults who engaged in high-intensity physical activity on 4 or more days per week exhibited a higher prevalence of traumatic dental injury compared to adults who did not exercise.

## Figures and Tables

**Table 1:** Characteristics of the participants according to the experience of traumatic dental injuries due to exercise.

Characteristics	Experience of traumatic dental injury				*p* Value
	No		Yes		
	Unweighted N	Weighted % (95% Cl)	Unweighted N	Weighted % (95% Cl)	
Age, years, mean (SD)	5,357	29.67 (0.13)	26	27.20 (1.18)	**<0.001**
Sex					**<0.001**
Male	2,177	98.9 (98.1–99.3)	20	1.1 (0.7–1.9)	
Female	3,180	99.8 (99.4–99.9)	6	0.2 (0.1–0.6)	
Education					**<0.001**
≤High school graduation, university withdrawal	1,618	99.3 (98.6–99.6)	11	0.7 (0.4–1.4)	
University or college in progress, completion, leave of absence	769	98.2 (96.2–99.1)	8	1.8 (0.9–3.8)	
≥University graduation	2,900	99.8 (99.5–99.9)	6	0.2 (0.1–0.5)	
Smoking status					0.132
No	3,154	99.5 (99.0–99.8)	11	0.5 (0.2–1.0)	
Yes	2,143	99.0 (98.3–99.5)	15	1.0 (0.5–1.7)	
Drinking status					0.831
No	186	99.4 (96.0–99.9)	1	0.6 (0.1–4.0)	
Yes	5,111	99.3 (98.9–99.6)	24	0.7 (0.4–1.1)	
Hypertension					0.508
No	5,176	99.3 (98.9–99.6)	24	0.7 (0.4–1.1)	
Yes	111	99.7 (97.5–100.0)	1	0.3 (0.0–2.5)	
Diabetes mellitus					**0.018**
No	5,244	99.4 (99.0–99.6)	24	0.6 (0.4–1.0)	
Yes	43	95.1 (72.3–99.3)	1	4.9 (0.7–27.7)	
Income (individual)					0.658
Lowest	1,270	99.3 (98.4–99.7)	7	0.7 (0.3–1.6)	
Middle low	1,353	99.5 (98.3–99.9)	3	0.5 (0.1–1.7)	
Middle high	1,336	99.5 (98.7–99.8)	6	0.5 (0.2–1.3)	
Highest	1,349	99.0 (97.7–99.6)	9	1.0 (0.4–2.3)	
Intense exercise					**0.045**
Never	3,492	99.5 (99.1–99.7)	15	0.5 (0.3–0.9)	
1 day a week	651	99.4 (96.0–99.9)	1	0.6 (0.1–4.0)	
2–3 days a week	726	99.3 (98.2–99.7)	5	0.7 (0.3–1.8)	
≥4 days a week	428	97.7 (94.3–99.1)	5	2.3 (0.9–5.7)	
Moderate exercise					0.23
Never	3,280	99.5 (99.0–99.7)	14	0.5 (0.3–1.0)	
1 day a week	565	99.2 (95.8–99.8)	2	0.8 (0.2–4.2)	
2–3 days a week	855	99.4 (98.5–99.8)	4	0.6 (0.2–1.5)	
≥4 days a week	597	98.4 (96.1–99.3)	6	1.6 (0.7–3.9)	

*Note*: Significant associations in bold. Continuous variables are expressed as mean (SD) and categorical variables as %. Values are presented as the unweighted number (*N*) (%). Weighted percent, 95% confidence interval (CI), and *p* value obtained by chi-squared test. The monthly household income was adjusted for the number of household members and classified into quartiles (average monthly household income/the number of household members): the lowest, lower middle, middle high, and highest. Analysis of variance for continuous variables and chi-squared test for categorical variables; SD, standard deviation.

**Table 2:** Multivariable association between frequency of exercise intensity and traumatic dental injury due to exercise.

Exercise intensity	Odds ratio (95% confidence interval)			
	Over four times a week	Two to three times a week	One time a week	Never (reference)
Intense exercise				
Model 1	**4.81 (1.55–14.89)**	1.48 (0.50–4.39)	1.19 (0.15–9.41)	1
Model 2	**3.16 (1.04–9.57)**	1.12 (0.36–3.49)	0.83 (0.11–6.43)	1
Model 3	**3.35 (1.03–10.86)**	1.20 (0.37–3.90)	0.91 (0.11–7.31)	1
Model 4	2.41 (0.67–8.63)	1.20 (0.36–3.94)	0.90 (0.12–6.79)	1
Moderate exercise				
Model 1	**3.11 (1.02–9.46)**	1.09 (0.33–3.62)	1.52 (0.25–9.38)	1
Model 2	2.38 (0.79–7.19)	1.04 (0.31–3.48)	1.25 (0.20–7.70)	1
Model 3	2.52 (0.79–8.08)	1.18 (0.35–4.01)	2.52 (0.79–8.08)	1
Model 4	1.91 (0.55–6.63)	1.27 (0.37–4.31)	1.43 (0.24–8.72)	1

*Note*: Significant associations in bold. Response variable: TDI due to exercise. Explanatory variable: intense exercise, moderate exercise. Model 1 was crude model. Model 2 was adjusted for age and sex. Model 3 was adjusted for age, sex, and income. Model 4 was adjusted for age, sex, income, and level of education. *Abbreviation*. TDI, traumatic dental injury.

**Table 3:** Characteristics of participants according to the frequency of intense exercise.

	Frequency of intense exercise, *N* (unweighted %)				
	Over 4 days a week	2–3 days a week	1 day a week	Never	*p* Value
Age, years, mean (SD)	28.39 (0.33)	29.74 (0.27)	29.59 (0.28)	29.82 (1.15)	**<0.001**
19–29	206 (11.0)	276 (14.3)	275 (13.5)	1,329 (61.3)	**0.003**
30–39	233 (7.9)	467 (15.7)	385 (11.9)	2,227 (64.5)	
Sex					**<0.001**
Male	252 (12.4)	409 (18.9)	4.8 (17.5)	1,139 (51.3)	
Female	187 (6.2)	334 (10.9)	252 (7.6)	2,417 (75.3)	
Education					**<0.001**
≤High school, university withdrawal	155 (10.3)	236 (15.5)	157 (9.2)	1,103 (65.0)	
University or college in progress, completion, leave of absence	94 (13.6)	117 (15.0)	116 (15.2)	458 (56.2)	
≥University graduation	187 (7.0)	389 (14.7)	385 (14.0)	1,987 (64.3)	
Income (individual)					**0.011**
Lowest	103 (8.8)	163 (12.9)	139 (11.1)	880 (67.2)	
Middle low	105 (8.7)	205 (16.4)	162 (13.2)	890 (61.8)	
Middle high	95 (8.3)	172 (14.0)	186 (14.7)	895 (63.0)	
Highest	132 (11.8)	197 (17.1)	170 (12.2)	858 (59.0)	
Smoking status					**<0.001**
No	218 (7.9)	368 (12.4)	317 (10.4)	2,297 (69.3)	
Yes	221 (11.0)	375 (18.0)	343 (15.2)	1,258 (55.8)	
Drinking status					0.415
No	12 (8.0)	23 (15.1)	14 (8.1)	141 (68.9)	
Yes	427 (9.4)	720 (15.0)	646 (12.8)	3,415 (62.8)	
Hypertension					0.917
No	425 (9.3)	728 (15.1)	645 (12.7)	3,473 (63.0)	
Yes	11 (10.0)	14 (12.2)	13 (12.9)	75 (64.8)	
Diabetes mellitus					0.245
No	433 (9.4)	736 (15.0)	649 (12.6)	3,522 (63.0)	
Yes	3 (4.6)	6 (12.2)	9 (23.7)	26 (59.5)	

*Note*: Significant associations in bold. Continuous variables are expressed as mean (SD) and categorical variables as %. Values are presented as the unweighted number (*N*) (%). Weighted percent, 95% confidence interval (CI), and *p* value obtained by the chi-squared test. The monthly household income was adjusted for the number of household members and classified into quartiles (average monthly household income/the number of household members): the lowest, lower middle, middle high, and highest.

## Data Availability

The raw data is available at https://knhanes.kdca.go.kr/knhanes/sub03/sub03_02_05.do. There are no limitations to accessing the data. Anyone can download it by registering their email address. Detailed data used to support the conclusions of this study are available upon request from the corresponding author.
